# Selenium intake, status, and health: a complex relationship

**DOI:** 10.1007/s42000-019-00125-5

**Published:** 2019-08-06

**Authors:** Margaret P Rayman

**Affiliations:** Department of Nutritional Sciences, Faculty of Health and Medical Sciences, University of Surrey, Guildford, GU2 7XH UK

**Keywords:** Selenium, Deficiency, Excess, Adverse health effects, Toxic elements, Dose, Speciation, Polymorphisms, Microbiota

## Abstract

Both selenium (Se) deficiency and excess are found in natural locations throughout the world, though Se excess can also be caused by supplementation with Se. Both have been associated with adverse health effects that have often been characterized by a U-shaped relationship. Some health effects, such as increased mortality, are associated with both low and high Se status. Certain people and populations are better able to tolerate low or high Se intake than others; there are a number of possible explanations for this fact. Firstly, it may relate to the presence of polymorphisms (SNPs) in genes that improve the ability to deal with a low or high Se intake. Secondly, high Se status, with apparent absence of toxicity and even beneficial effects, can be found in populations exposed to toxic elements that are known to interact with Se, forming complexes in some cases. Thirdly, beneficial and harmful effects of Se depend on Se dose and form (speciation); for instance, at a high dose, selenomethionine (SeMet) has toxic effects that are mediated by metabolism to selenols/selenolates that can redox-cycle, generate superoxide radicals and react with thiols/diselenides to produce selenyl sulphides/disulphides. Finally, it is possible that exposure to a high Se intake from birth or from a very young age may alter the composition of the gut microbiota in such a way that excess Se is more readily excreted, thus reducing its toxicity.

## Introduction

The highly insightful observation made by Confucius (551 to 479 BC), “Excess and deficiency are equally at fault”, is as relevant today as it was 2500 years ago, and it applies to almost all nutrients, though it is particularly apposite to selenium (Se).

Se intake is extremely variable across the world [1] owing to a number of factors, including the Se content of the soil in which crops and fodder are grown, Se speciation, soil pH and organic-matter content, and the presence of ions that can complex with Se [2]. This fact enables the effects of both Se deficiency and excess to be observed in the natural world.

## Effects of Se deficiency

Some adverse health conditions associated with Se deficiency are listed in Table 1.

**Table 1:** Conditions that have been associated with Se deficiency [3]

Condition	Reference
Keshan disease	[2]
Kashin-Beck disease	[4]
Increased viral virulence	[5]
Increased mortality	[6]
Poorer immune function	[7]
Problematic fertility/reproduction	[3]
Thyroid autoimmune disease	[8]
Cognitive decline/dementia	[9–11]
Type 2 diabetes	[12]
Prostate cancer risk	[13]
Colorectal cancer risk *in women* (seen in EPIC)	[14]

A further example that is less well known relates to the reduction in risk of incident tuberculosis (TB) in HIV-infected subjects who were treated with Se. Because HIV is an immune-deficiency syndrome, those with this disease commonly suffer from other infections, such as TB, particularly as the condition progresses [15]. In a 24-month, four-arm, randomized, controlled trial (RCT) in 878 HIV-infected adults in Botswana, who were randomized to Se, multivitamins, Se+multivitamins or placebo, treatment with 200 μg Se+multivitamins significantly reduced the risk of immune decline and morbidity, while multivitamins alone and Se alone had no effect [16]. Importantly, Se vs. placebo significantly reduced the risk of incident TB: hazard ratio (HR) 0.20, 95%; confidence interval (CI): 0.04, 0.95; *P* = 0.043. When the Se and Se+multivitamin arms were combined, the risk of TB was also reduced: HR 0.32, 95% CI: 0.11, 0.93; *P* = 0.036 [15]. This is an important finding in relation to HIV outcome, particularly in sub-Saharan Africa, where TB can often be fatal.

## Effects of Se excess

The effects of Se excess are probably less well known. Apart from some occasional cases of overdose where people have ingested wrongly formulated supplements [17], Se excess has mostly been observed in RCTs, where doses of 200 μg/day or more of Se have been given for a substantial period of time (see Table 2).

**Table 2:** Health risks significantly related to selenium excess

Health condition	Reference
Selenosis (Se toxicity)	[2, 17]
Alopecia (seen in SELECT)	[18]
Dermatitis (seen in SELECT)	[18]
Non-melanoma skin cancer (seen in NPC trial)	[19]
Increased mortality	[6] [20]
Type 2 diabetes (seen in the NPC trial)	[21]
Increased prostate cancer risk (seen in SELECT)	[22]

## Adverse health effects associated with, or caused by, both Se deficiency and excess: Se and mortality

It is of note that some health conditions are associated with both Se deficiency and excess, i.e. increased mortality, type 2 diabetes and increased prostate cancer risk [6, 12, 13]. Increased mortality is seen at both low and high plasma Se concentrations. This was first demonstrated by the Guallar group at Johns Hopkins School of Public Health, Baltimore, USA, which examined data from the US Third National Health and Nutrition Examination Survey. Serum Se was measured in 13,887 adult participants who were then followed up for mortality for up to 12 years [6] and subsequently for a further 6 years [3]. Mortality showed a U-shaped association, reaching a minimum at a serum Se concentration of 135 μg/L.

**Fig. 1:**
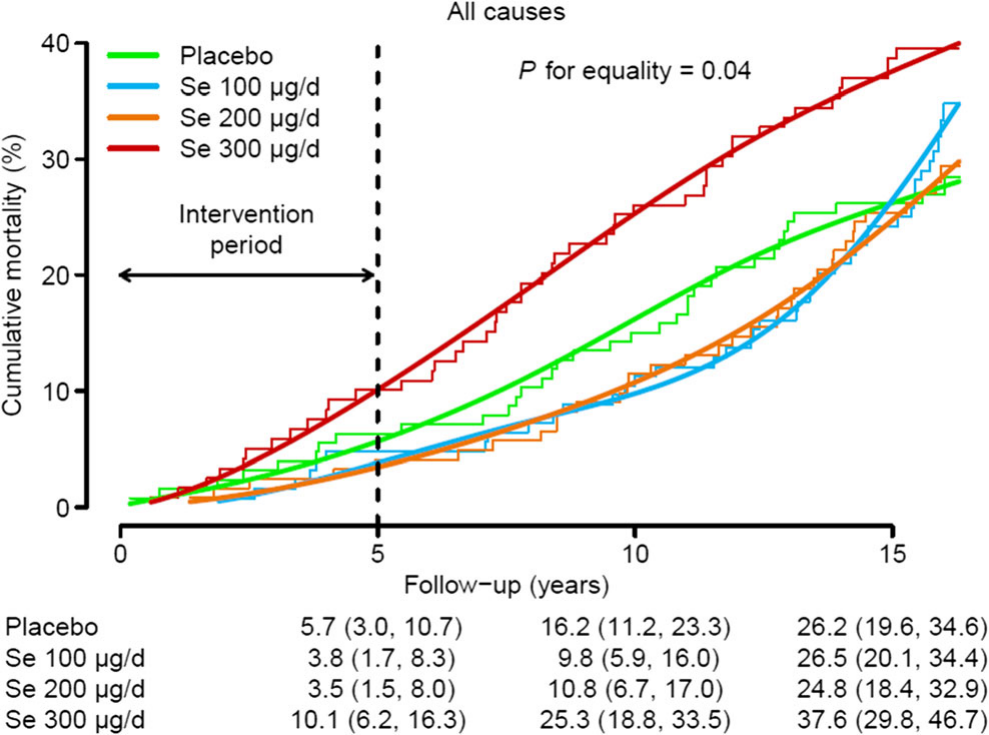
Cumulative mortality from all causes over time by treatment group. Non-parametric cumulative mortality curves (step functions) were estimated via the Kaplan-Meier method and compared with results generated by the generalized Wilcoxon test. Parametric cumulative mortality curves (smooth lines) were estimated from spline-based parametric survival models with treatment-specific log cumulative hazards parameterized as natural cubic splines of log time with knots at the 33th and 67th percentiles of the uncensored log-time distribution. Cumulative mortality estimates (95% CIs) at 5, 10 and 15 years of follow-up by treatment group were obtained from spline-based parametric survival models. Se denotes selenium [20] (published with permission of FRBM)

Increased mortality was seen at the highest dose level of an RCT of Se in the Danish PRECISE trial, a randomized, double-blinded, placebo-controlled, clinical trial with four groups [20]. Participants were 491 male and female volunteers aged 60–74 years who were randomly assigned to treatment with 100, 200 or 300 μg Se/day as Se-enriched yeast or placebo yeast for 5 years from randomization in 1998–1999 and were followed up for mortality for a further 10 years. In an intention-to-treat analysis, the HR (95% CI) for all-cause mortality comparing 300 μg Se/day with placebo was 1.62 (0.66, 3.96) after 5 years of treatment and 1.59 (1.02, 2.46) over the entire follow-up period [20]. The relationship between cumulative mortality from all causes over time by treatment group in the Danish PRECISE RCT is shown in Fig. 1. The results of this study warn that a 300-μg/day dose of Se (as Se yeast) taken for 5 years in a country with moderately low Se status can increase all-cause mortality 10 years later.

**Fig. 2:**
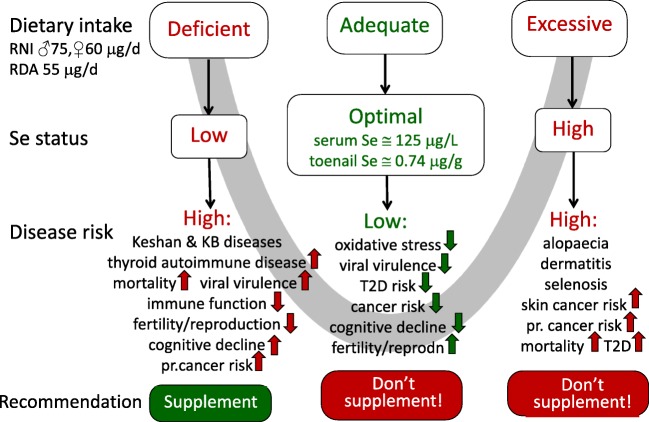
U-shaped relationship between Se status and disease risk (for references, see [3])

The relationship between Se intake/status and mortality is an excellent example of the U-shaped association between Se and health. Figure 2 illustrates that relationship, additionally including data referred to in Tables 1 and 2 [23].

## Complexities in the relationship between Se intake and health effects: factors that need to be considered

Though the results of the Danish PRECISE trial show excess mortality on long-term (5 year) exposure to 300 μg Se/day as Se yeast of a population experiencing much lower Se exposure (plasma-Se concentration at baseline, 89 μg/L, that rose to 284 μg/L after 5 years) [20], it is notable that population groups with long-term high-Se exposure appear to cope with a Se intake or status of the same magnitude as in that trial without suffering adverse effects, e.g. Inuit of Greenland and Northern Canada, populations of the Brazilian Amazon, and some local Chinese populations. There are a number of possible reasons for this, as outlined below.

1. It appears that some people and/or populations are better able to tolerate low or high Se intake than others owing to the presence of polymorphisms (SNPs) in genes that affect their ability to deal with a low or high Se intake. This has been best demonstrated in relation to deficient intake. The allele frequency of SNPs in several selenoprotein genes changes in response to differences in environmental Se [24], e.g. SNPs in:
  (i) Selenoprotein genes, *GPX1*, *DIO2*, *DIO3*, *SELENOS*, *SELENOM*
  (ii) Genes involved in Se/Sec regulation of *CELF1*, *SPS2, SEPSECS*, *ELAVL1*, *Ser-tRNA*^*Sec*^ and *SELENOP*
  (iii) Genes involved in excretion/detoxification of Se, such as *INMT (indolethylamine N-methyltransferase).*

In relation to the third point, Se is excreted by being metabolized to methylated Se products in breath or urine and to urinary selenosugars [25]. Individuals with the AA or AG *INMT* genotype can metabolize Se to trimethyl-selenonium ion (TMSe) in the urine, thereby excreting more Se and potentially reducing toxicity [26]. The frequency of TMSe production varies markedly between populations and individuals [26].

2. High Se status, with apparent absence of toxicity and even beneficial health effects, can be found in populations exposed to toxic elements, such as arsenic, cadmium and mercury, all of which are known to interact with Se. Sequestration in inert conjugates that can be excreted has been suggested as accounting for this. Various structures have been proposed for a complex between mercury and selenium or selenoprotein P (SELENOP), such as MeHg-Se-SELENOP [27] and (Hg-Se)n]m-SELENOP [28]. A complex of arsenic and Se [(GS)2AsSe] has been detected in rabbits [29] while a cadmium-Se complex with a molar ratio of 1:1 which was able to bind to SELENOP, has been shown to form in vitro [30].
  (i) High mercury content is found in the diet of the Inuit in Greenland and Nunavik (Northern Quebec) as a result of consumption of top predator marine mammals and fish [31]. In a study of 852 Inuit adults who participated in the 2004 Nunavik Inuit Health Survey, up to 50% of plasma mercury was associated with SELENOP [32]. In the International Polar Year Inuit Health Survey (IHS) of Inuit in Canada conducted in 2007 and 2008, geometric mean blood Se and total Hg were 319.5 μg/L and 7.0 μg/L, respectively [33]. High Se exposure was shown to lower the odds ratios for hypertension, stroke, and myocardial infarction associated with mercury exposure [33]. In the same study, participants with stroke had lower blood Se (geometric mean: 260 μg/L vs. 319 μg/L) and dietary Se (144 μg/day vs. 190 μg/day) than individuals without stroke [34].

High levels of dietary mercury are also found in the Brazilian Amazon, notably in communities of the Tapajós River [mean intake (SD) 0.92 (0.89) μg/kg/day] [35]. The dietary Se intake in these villages ranges from normal to very high owing to the consumption of large amounts of Brazil nuts, chicken, game and certain fish species [36]. In participants recruited from 12 communities along the Tapajós River, median blood and plasma Se were 228 (range 103–1500) μg/L and 135 (range 54–913) μg/L, respectively [36]. Although blood and plasma concentrations exceeded concentrations considered toxic [blood Se: 1000 μg/L (US EPA 2002)], no signs of Se toxicity (dermal or breath) or sentinel symptoms (gastrointestinal disorders and motor/sensory deficits) were found, despite very high Se status in some individuals [36]. Interestingly, only three of 407 participants reported diabetes.

(ii) Rice easily takes up arsenic and elevated exposure is frequent in areas of Bangladesh, India, China and Thailand [37]. An antagonistic relationship between Se and arsenic was first observed in 1938 when it was discovered that Se-poisoned rats could be treated with arsenic [38]. Antagonism between arsenic and Se, whereby each reduces the toxicity of the other, has been well documented in animal models [39].
(iii) Cadmium, a potent pro-oxidant, accumulates in the kidney, increasing oxidative stress [40]; the demand for Se in the kidney may increase with higher cadmium exposure, as Se is required for the expression of the antioxidant enzyme, GPX3, which is produced in the kidney [41]. High cadmium was found in the Enshi area of China known for Se intake in the toxic range [42].

**Fig. 3:**
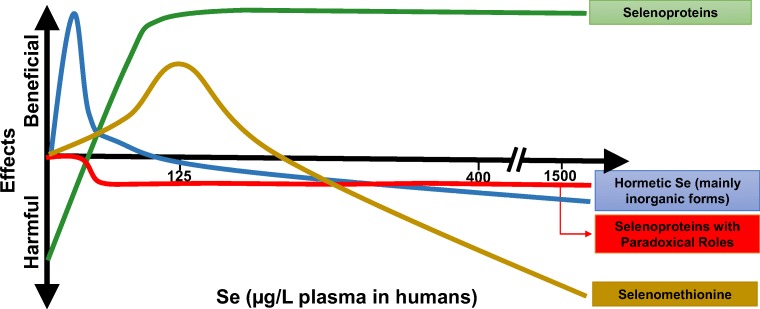
Beneficial and harmful effects of Se depending on dose and form (speciation). It is proposed that Se has hermetic effects, shown by the blue line [43]. Selenoproteins with beneficial health effects are shown as a green line, while selenoproteins with paradoxical roles that appear to have harmful effects are shown as a red line [43–45]. As Se intake increases, particularly if the main source is SeMet, SeMet will increasingly replace methionine in body proteins, where its turnover is exceptionally slow; SeMet has toxic effects that are explained in the text (figure created with assistance from Wen-Hsing Cheng, Mississippi State University, Mississippi State, USA)

3. Beneficial and harmful effects of Se depend on Se dose and form (speciation) (see Fig. 3). It has been proposed that Se may have hermetic effects, i.e. a biphasic dose response to an agent characterized by a beneficial effect at a low dose and an inhibitory or toxic effect at a high dose [43]; this can be represented by the U-shaped response to increasing Se intake/status, as observed.
  While selenoproteins are generally considered to have beneficial health effects, there are selenoproteins with paradoxical roles with potentially harmful outcomes [43], e.g. GPX1, implicated in insulin resistance [44] and SELENOP, associated with type 2 diabetes [45].

In relation to Se dose, as Se intake increases, particularly if the main source is selenomethionine (SeMet) or Se yeast (60% of which is SeMet [46]), SeMet will increasingly replace methionine in body proteins, where its turnover is exceptionally slow (whole-body turn-over time, 363 days) [47]. Of course, SeMet can also be metabolized to selenide, whereby it can act as a source of selenoproteins. However, SeMet has toxic effects that are mediated by metabolism to selenols/selenolates that can redox-cycle, generate superoxide radicals, and react with thiols/diselenides to produce selenyl sulphides/disulphides [20]. The latter can cause protein aggregation, inactivation of transcription factors, disruption of redox-regulated cell signaling, and endoplasmic reticulum (ER) stress [20]. It should be noted that Se, as sodium selenite, would be metabolized to hydrogen selenide, used for the generation of selenoproteins or excreted, and would not build up in the body in the same way as SeMet [48].

4. About one quarter of all bacteria express selenoproteins and therefore require Se for optimal growth [49]. Gut microorganisms are sensitive to trace elements and some require Se for normal metabolic functions [49]. In metabolizing Se, they produce volatile methylated Se compounds, such as dimethyldiselenide from SeMet, in the intestinal tract that are excreted, thus protecting the host from toxicity due to high Se exposure [50]. It is possible that exposure to high Se intake from birth or from a very young age alters the composition of the gut microbiota in such a way that excess Se is more readily excreted. Of course, this use of Se by microorganisms decreases the availability of Se for the expression of host selenoproteins, resulting in increased requirement of the host for Se [49].

## Conclusion

The concept of the U-shaped relationship between Se intake/status and its health effects, while useful, is clearly somewhat simplistic. We need a better understanding of the subtle factors outlined above that affect the Se requirements for optimal health, which vary by population and, indeed, between individuals. It would be interesting, for instance, to investigate whether the gut microbiome differs significantly in high vs. moderate consumers of Se. Se will still keep us guessing for some time to come.
